# The effect of a national quality improvement collaborative on prehospital care for acute myocardial infarction and stroke in England

**DOI:** 10.1186/1748-5908-9-17

**Published:** 2014-01-23

**Authors:** Aloysius Niroshan Siriwardena, Deborah Shaw, Nadya Essam, Fiona Jayne Togher, Zowie Davy, Anne Spaight, Michael Dewey

**Affiliations:** 1 East Midlands Ambulance Service NHS Trust (EMAS), Cross O’Cliff Court, Lincoln LN4 2HN, England; 2 University of Lincoln, Brayford Campus, Lincoln LN6 7TS, England; 3 Freelance statistician, Salisbury, England

**Keywords:** Quality improvement, Performance measures, Prehospital care, Team training, Audit and feedback, Interrupted time series design

## Abstract

**Background:**

Previous studies have shown wide variations in prehospital ambulance care for acute myocardial infarction (AMI) and stroke. We aimed to evaluate the effectiveness of implementing a Quality Improvement Collaborative (QIC) for improving ambulance care for AMI and stroke.

**Methods:**

We used an interrupted time series design to investigate the effect of a national QIC on change in delivery of care bundles for AMI (aspirin, glyceryl trinitrate [GTN], pain assessment and analgesia) and stroke (face-arm-speech test, blood pressure and blood glucose recording) in all English ambulance services between January 2010 and February 2012. Key strategies for change included local quality improvement (QI) teams in each ambulance service supported by a national coordinating expert group that conducted workshops educating staff in QI methods to improve AMI and stroke care. Expertise and ideas were shared between QI teams who met together at three national workshops, between QI leads through monthly teleconferences, and between the expert group and participants. Feedback was provided to services using annotated control charts.

**Results:**

We analyzed change over time using logistic regression with three predictor variables: time, gender, and age. There were statistically significant improvements in care bundles in nine (of 12) participating trusts for AMI (OR 1.04, 95% CI 1.04, 1.04), nine for stroke (OR 1.06, 95% CI 1.05, 1.07), 11 for either AMI or stroke, and seven for both conditions. Overall care bundle performance for AMI increased in England from 43 to 79% and for stroke from 83 to 96%. Successful services all introduced provider prompts and individualized or team feedback. Other determinants of success included engagement with front-line clinicians, feedback using annotated control charts, expert support, and shared learning between participants and organizations.

**Conclusions:**

This first national prehospital QIC led to significant improvements in ambulance care for AMI and stroke in England. The use of care bundles as measures, clinical engagement, application of quality improvement methods, provider prompts, individualized feedback and opportunities for learning and interaction within and across organizations helped the collaborative to achieve its aims.

## Background

The annual incidence of acute myocardial infarction (AMI) in the United Kingdom (UK) is approximately 268,000 cases [1], two-fifths of which result in sudden death. Stroke and transient ischaemic attack (TIA) has an incidence of 150,000 per year in the UK [2]. AMI and stroke are the most common cause of death in the UK and a major cost to the economy. Ambulance services are usually the first healthcare response for these conditions.

Early recognition and treatment of AMI and stroke has the potential to improve patient experience, prevent deaths and reduce long-term ill-health and disability [3,4]. Recent studies of ambulance service indicators in England have shown variations and shortfalls in prehospital care for AMI and stroke [5], making this a priority for improvement [6]. Such variations are often due to clinician, patient or organizational factors [7,8].

Quality Improvement Collaboratives (QICs) are multiorganizational, multiprofessional initiatives in which improvement and clinical experts, using structured activities, engage clinicians to effect improvement in a specific area of practice [9]. They have been used for over a quarter of a century in acute and primary care but, despite some positive evidence for their effectiveness [10], until now there have been few published QICs involving ambulance services [11].

The Ambulance Services Cardiovascular Quality Initiative (ASCQI) aimed to evaluate the effectiveness of a national QIC for implementing more reliable, high-quality prehospital care for AMI and stroke in England.

## Methods

### Design and setting

We used mixed methods with an interrupted time series design to determine the effect of the interventions in each trust and the collaborative as a whole, and a multiple case study approach [12] combining questionnaires, semi-structured interviews, observation of collaborative meetings, document analysis and pattern matching with time series data to understand how and why the collaborative led to improvement [13]. The results of the interrupted time series and key findings from the multiple case study design are presented here.

The interrupted time series design was used to investigate the effect of the QIC on change in delivery of care bundles for AMI and between January 2010 and February 2012.

We included all twelve publicly funded National Health Service (NHS) ambulance organizations (trusts) in England comprising 22,117 ambulance clinicians providing urgent prehospital care to the whole population. Each trust covers a large geographical area and operates widely dispersed clinical teams where clinicians work under time pressures and respond to urgent conditions.

The study was approved as a Quality Improvement Project by participating ambulance services and independent review was undertaken by the University of Lincoln School of Health and Social Care Ethics Committee. The Engaging Ambulance Clinicians in Quality Improvement: Questionnaire Study was approved by the National Research Ethics Service Committee East Midlands – Nottingham 1 (REC reference: 10/H0403/83).

### Quality improvement collaborative (ASCQI)

#### Collaborative background and aims

Prior to this study, in 2007, the National Ambulance Services Clinical Quality Group (NASCQG) was formed to measure clinical quality in ambulance services. The aim was to widen assessment beyond the previous primary indicator of response time. Clinical indicators were developed for five conditions, including AMI and stroke, which showed wide variations in care when they were introduced [5].

In 2009, we established a national QIC (ASCQI) funded by the Health Foundation, supported by Chief Executive Officers (CEOs) and Medical Directors of ambulance trusts, and reporting to the NASCQG. The QIC consisted of a national expert team that supported local QI teams in each ambulance service. Each ambulance service had a QI lead who sat on the NASCQG and led the local QI team within their service. The national expert team included two co-leads (one service/academic QI expert and one clinical expert), a project manager, QI leads from two other services, a QI fellow (QIF) from one service, a data analyst, two facilitators (one QI expert and one to support the team and its members), and administrative support.

**Figure 1:**
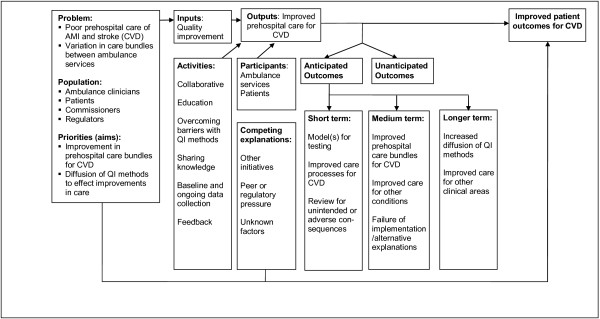
Logic model for Ambulance Services Cardiovascular Quality Improvement Initiative.

We used a logic model (Figure 1) and programme theory to develop the QIC. This was based on a clear aim and measurement strategy, teamwork, national (external agent) support, local organizational support and multiple mechanisms for sharing learning [14].

#### Improvement measures

Although previous research has found that paramedic diagnosis of stroke (based on clinical presentation) [15] and AMI (based on clinical presentation and ECG) [16] is very accurate, we were interested in how paramedics managed patients with suspected AMI and stroke and how this could be improved. This was the critical issue for the quality of service provided, rather than whether the correct management was carried out only in those subsequently confirmed with these conditions.

A pre-existing care bundle for patients with a prehospital diagnosis of AMI and stroke was adopted as the key improvement measure (Table 1). A care bundle is a set of indicators, measured according to whether each was delivered for every eligible patient (unless there was a valid exception). For AMI this included aspirin, glyceryl trinitrate [GTN], pain assessment and analgesia, and for stroke, the face-arm-speech test, blood pressure and blood glucose recording [17]. These measures were based on evidence of best practice [18,19] which had been translated into guidance adopted by UK ambulance services [20].

**Table 1:** Care bundles for AMI and stroke

***Acute myocardial infarction (AMI)***	
M1	Aspirin
M2	GTN
M3	Initial pain score
M4	Final pain score (assumed intervention)
M5	Analgesia given
M5i	Morphine
M5ii	Morphine and/or Entonox
*M1 + M2 + M3 + M4 + M5*	*AMI Care bundle*
***Stroke***	
S1	FAST assessment recorded
S2	Blood glucose recorded
S3	Blood pressure recorded
*S1 + S2 + S3*	*Stroke care bundle*

#### Improvement activities

The local QI team in each ambulance trust set up collaborative workshops led by a QIF at each participating trust usually supported by the project manager or a QIF from another service. Workshops involved ambulance clinical staff including emergency care practitioners, paramedics, technicians, and emergency care assistants.

The collaborative workshops used QI methods that had been tried and tested in other healthcare settings including: analysis of barriers and facilitators to improvement using patient interviews and focus groups of staff [8]; process maps, cause-and-effect (fishbone or Ishikawa) diagrams, and critical-to-quality trees to understand how best to improve processes; and plan-do-study-act cycles to test process redesign.

Interventions, designed to improve care were initially tested in small areas of individual trusts to address the issues or barriers they identified [21]. If particular initiatives proved effective in those areas, these were spread more widely across a whole trust and shared with other trusts.

We encouraged services to develop and test contextually driven combinations of interventions, which formed a complex intervention, specific to each trust. Interventions included awareness days, information posters, ‘reminder’ pens and key fobs, educational work packages, presentations and fact sheets, assessment tools developed and equipment bags redesigned to support the delivery of the care bundles. Most trusts implemented aide memoires and checklists to prompt providers to complete the care bundles as well as mechanisms to ensure timely individualized feedback to frontline staff. One trust also implemented a financial incentive scheme linked to their feedback system.

Knowledge of the interventions and their effect in each service was shared across services using national group meetings (three meetings during the course of the QIC), coordinating meetings, monthly teleconferences for QIFs, and an electronic repository to store and disseminate information.

#### Feedback

Eleven of the 12 trusts completing the QIC submitted data on performance for individual indicators, care bundles, and the age and gender of patients included in the study. QIFs were also asked to report monthly on the interventions that took place through a written feedback form, and this information was also shared and discussed during monthly teleconferences with the lead QIF who also reported back to the expert team.

Control charts of weekly performance data (from Monday to Sunday) consisting of 25 records (or all cases if there were fewer than 25) from each trust were used to analyze changes in performance over time and these were fed back to local QI teams every three months. Control charts were annotated with interventions implemented by each service to understand which interventions were effective and estimate the size of their effect [22].

#### Data collection and analysis

We used two methods performed independently to measure outcomes.

First, we established baseline rates for care bundle performance for ambulance services. This was part of the National Ambulance Clinical Performance Indicator (NCPI) project that started before the collaborative began. We used measurement methods developed for ambulance clinical indicators [23] that involved collecting samples of up to 300 cases during one month from each organization, assessing the indicator in these cases and comparing this across services using funnel plots (Additional file 1: Figures S4 and S5). We showed baselines rates overall of 43% for AMI (in May 2008) and 83% (in July 2009) for stroke. The same method was used to show rates across services towards the end of the QIC.

Second, during the QIC we also collected and analyzed AMI and stroke data weekly. We used records generated during each week for the first 25 patients with AMI and stroke from each trust. This number was chosen based on average numbers of cases of AMI per week from a pilot data collection [5]. Data were collected prospectively and plotted weekly from January to June 2010 to ensure a sufficient period to establish baseline variation and secular trends. We continued collecting data weekly from 1 July 2010 to the end of the QIC on 28 February 2012.

The care bundle was defined as having been delivered if all the elements were undertaken: if an element was refused or otherwise contraindicated that element was treated as undertaken and the bundle was counted as delivered if all the other elements were carried out. Data collection, accuracy, and completeness were previously validated and refined as part of the NCPI project [23].

We conducted a preliminary analysis which showed a non-linear relationship between patients’ age and receipt of the care bundle, and we therefore used a logistic regression with three predictor variables: time (measured in weeks but presented per four weeks), gender, and a non-linear effect of age using a spline function, to predict receipt of the care bundle as a binary outcome (yes/no). The analysis over the whole time period of the QIC provided a model responsive either to gradual change over the time course or to a sudden change occurring at an unspecified point during the period in question.

The model was fitted separately to each trust and the coefficient for time and its standard error were then extracted from each fit and plotted using a forest plot. In this plot, the central estimate was plotted as a square with area proportional to the amount of information provided by that trust and a line representing the 95% confidence interval. For reference, a summary value was computed using a fixed effects model using the package Metafor [24]. Although in systematic reviews random effects models are often chosen especially when there is substantial heterogeneity, in this case the intention was to summarize the trusts involved rather than to generalize to a population of trusts and so the use of a fixed effects model was deemed more appropriate [25].

## Results

All twelve English ambulance services originally agreed to participate in the QIC. One trust (trust six) formally withdrew in January 2012 due to conflicting pressures.

**Figure 2:**
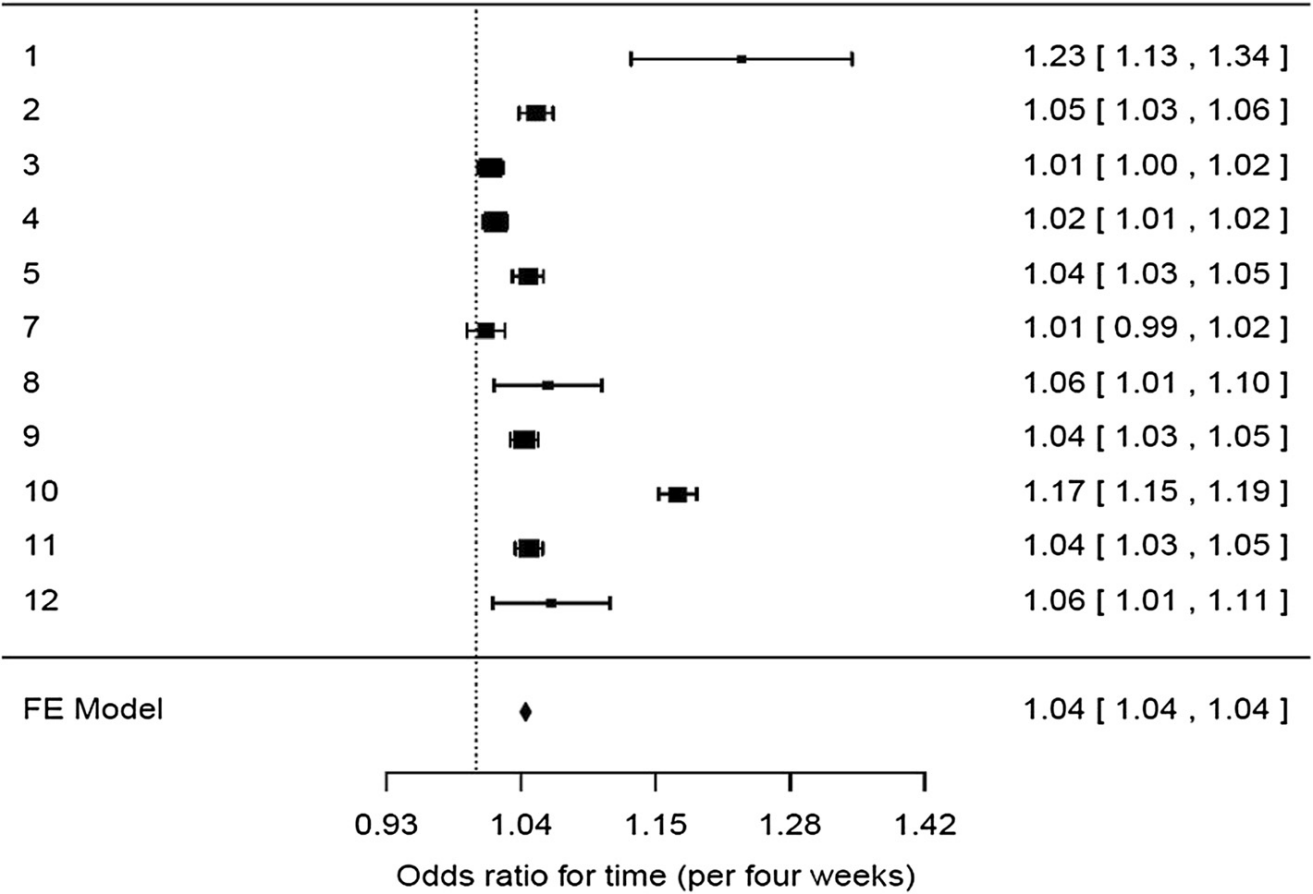
**Forest plot showing for each trust the odds ratio for receiving the AMI care bundle with time as a continuous variable.** The odds ratios come from a logistic model using time, gender, and age to predict receipt of the care bundle as a binary outcome (yes/no).

**Figure 3:**
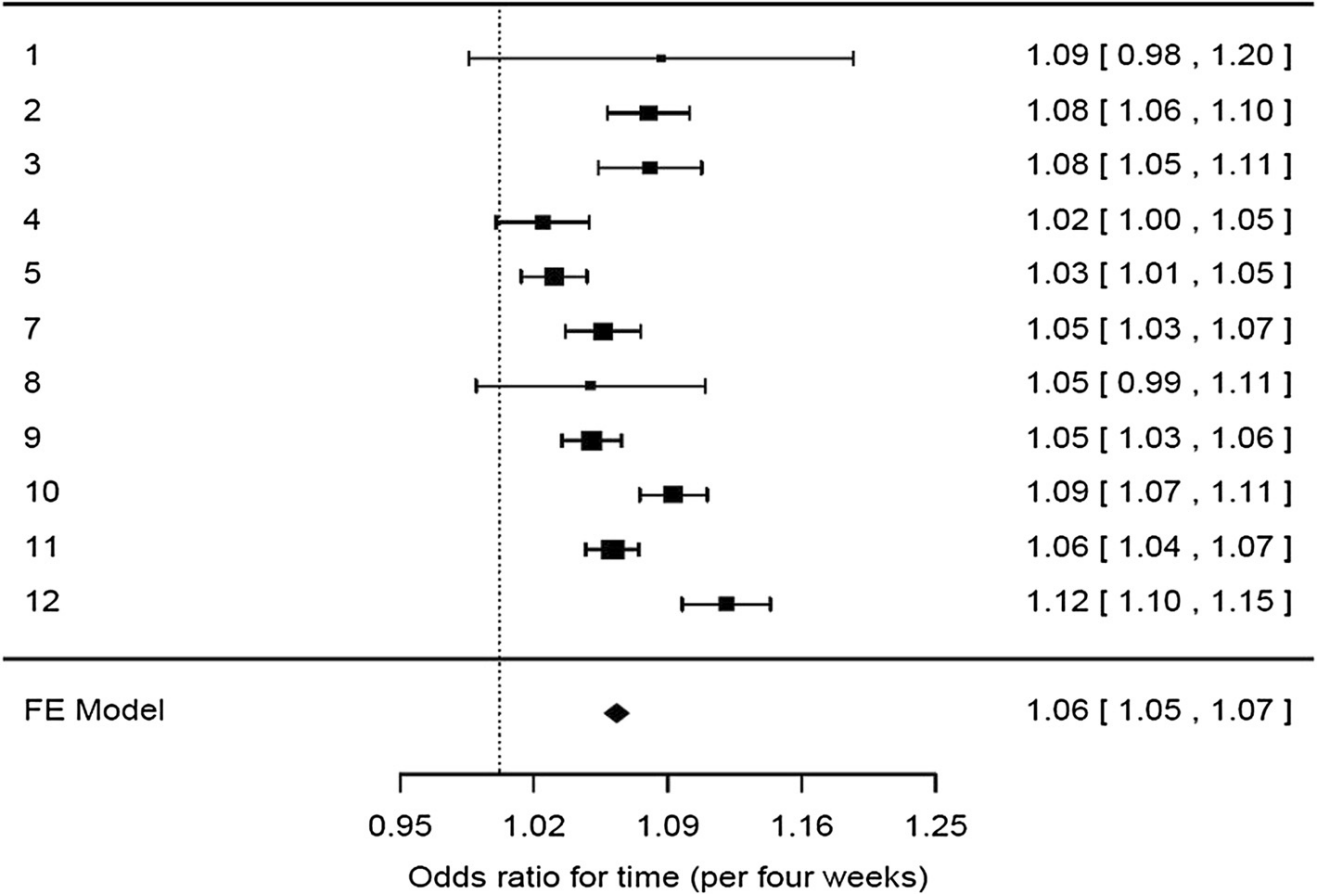
**Forest plot showing for each trust the odds ratio for receiving the stroke care bundle with time as a continuous variable.** The odds ratios come from a logistic model using time, gender, and age to predict receipt of the care bundle as a binary outcome (yes/no).

We included 19,446 patients with AMI and 25,373 patients with stroke in the analysis. The effect of time was for universal improvement for all trusts for AMI and stroke despite considerable heterogeneity; some trusts made substantial changes in performance compared with others (Figures 2 and 3).

The forest plots show the results from the model using time as a continuous variable. The confidence intervals demonstrated significant effects, except for trusts three and seven for AMI (Figure 2) and trusts one, four, and eight for stroke (Figure 3). There were statistically significant improvements in nine (of 12) participating trusts for the AMI care bundle (OR 1.04, 95% CI 1.04, 1.04) and eight (of 12) for the stroke care bundle (OR 1.06, 95% CI 1.05, 1.07). Ten of 12 trusts showed a significant improvement in either the AMI or stroke care bundle, and seven (of 12) showed significant improvements for both AMI and stroke.

The improvements, where these occurred, were due to reduction in variation and shifts in performance following the improvement efforts (Additional file 1: Figures S8–S11) rather than reflecting a secular trend or gradual adoption of care bundles.

The overall charts show wide variation from January to June 2010, *i.e.*, before improvement activities began, with a shift in care from November 2010 and a further shift in care from November 2011 (Additional file 1: Figures S12 and S13). Reductions in variation and shifts in care occurred initially after improvement activities began in most trusts.

National clinical indicator measures showed independently that overall performance for the care bundle for AMI increased nationally in England from 43 to 79% and for stroke from 83 to 96% following the QIC (Additional file 1: Figures S4–S7).

Although various combinations of interventions were used to improve care, tailored according to analysis of barriers in each trust, the two interventions most often used by successful services were provider prompts (checklists) and clinical feedback to frontline staff (see key interventions column labelled ‘Checklists, prompts or aide-memoires in Table 2). These interventions were associated with significant changes in the control chart data, which also meant they were shared more rapidly across services.

**Table 2:** Effect of performance on care bundles for AMI and stroke and key interventions used in English ambulance services 2010-2012

**Ambulance service**	**Effect on performance**				**Key interventions**		
	**AMI**	**Stroke**	**AMI or stroke**	**Both AMI and stroke**	**Checklists, prompts or aide-memoires**	**Individual feedback**	**Group feedback**
**1**	Yes	No	Yes	**No**	Yes	No	Yes
**2**	Yes	Yes	Yes	Yes	No	No	No
**3**	No	Yes	Yes	**No**	No	No	No
**4**	Yes*	No	Yes	Yes	No	No	No
**5**	Yes	Yes*	Yes	Yes	Yes	No	No
**6**	-	-	-	**No**	-	-	-
**7**	No	Yes	Yes	**No**	Yes	No	No
**8**	Yes*	No	Yes	**No**	Yes (late)	No	Yes
**9**	Yes	Yes	Yes	Yes	Yes	Yes	No
**10**	Yes	Yes	Yes	Yes	Yes	Yes	Yes**
**11**	Yes	Yes	Yes	Yes	Yes	Yes	No
**12**	Yes	Yes	Yes	Yes	Yes	No	Yes

*Odds ratio 1.01-1.03 (compared to OR ≥1.04 for other trusts where greater improvement occurred).

**With group financial incentives to ambulance stations.

## Discussion

### Summary

This was the first large-scale national prehospital QIC involving ambulance services worldwide. Eleven of the twelve English trusts completed the QIC and all showed significant improvement in either stroke or AMI care bundle; seven demonstrating significant improvements for both. The QIC achieved its aim of applying quality improvement methods to improve care for patients with AMI or stroke presenting to ambulance services in England. We showed that QICs can be successful in the context of prehospital urgent care through engagement of staff in the use of quality improvement methods and by providing individual feedback to frontline clinicians.

### Strengths and weaknesses

The strengths of the study included the national scale and the mixed methods design which included interrupted time series, multiple case studies and pattern matching linking these to understand the improvements that occurred and how these came about.

Because this was a national collaborative we were not able to exclude or control for other factors which may have reduced variation or increased performance. We were not aware of such factors operating at a national level during the time course of the QIC. If there had been an external factor causing change to the care bundles we would have expected change to occur broadly simultaneously in the trusts and to approximately the same extent. As the results we have presented show, this did not happen: trusts changed at different times and some did not change very much, if at all.

We could not account for comorbidity or demographic variables other than age or gender that could affect care, including socioeconomic deprivation, ethnicity, and rurality. Despite these limitations, we feel the lessons from this QIC are generalizable to other prehospital services in the developed world, and other areas of urgent and emergency care.

### Determinants of success

A systematic review of QICs suggested that possible factors contributing to empirical success of QI collaboratives were often only weakly predictive and inconsistent between studies; even factors that were identified, such as teamwork being facilitated or collaborative support being provided were linked to short term success only [26]. Schouten *et al.* developed an instrument based on literature and opinions of QI experts which suggested that three factors, ‘sufficient expert team support,’ ‘effective multidisciplinary teamwork,’ and ‘helpful collaborative processes’ may be key to effectiveness [27]. Another instrument, developed by Dückers *et al.* suggested that ‘organizational support,’ ‘team organization,’ and ‘external change agent support’ were important factors for success [14].

This QIC was designed to provide an environment that supported quality improvement. This was largely through engaging staff, particularly frontline staff at various levels of seniority, to share quality improvement expertise and experience at both local and national levels, and by providing reliable information about improvement throughout [28]. Engaging and involving frontline staff from project inception encouraged them to take ownership of and lead the changes that took place. Engagement also engendered positive responses from colleagues towards the changes that local collaborative members implemented [29].

Local teams, supported by local leaders and the national expert team, were able to develop greater skills and knowledge of QI methods in a practical and meaningful way to them through the QIC. We found frontline staff willing to try these new methods and to adopt the suggested changes because they understood these were a way of improving patient care, rather than arbitrary response time targets being met [5]. Some participating trusts reported that they had adapted these methods to improve the delivery of care for other clinical conditions, such as asthma, suggesting there were ‘spill-over’ effects’ from their learning about QI for AMI and stroke to other conditions.

The nature of the feedback provided through annotated control charts was also reported to be helpful in bringing about change. For example, ambulance services tended initially to use education and passive information dissemination but, because these approaches did not bring about change in the annotated control charts, these were superseded in more successful trusts by active methods such as provider prompts and individualized or team feedback. This resulted in significant shifts in performance in control charts where such methods were adopted. Less successful trusts were characterized by lack of engagement or delays (*e.g.*, trusts four and eight) in implementing the QIC process.

The detailed picture was more complicated. Trusts two and four did not report using provider prompts and individualized or team feedback interventions but did show improvement in AMI and stroke for trust two, and albeit only a small improvement for trust four. Furthermore, other trusts that used only one of these interventions (*e.g.*, trusts seven and eight) did not show consistent improvement. This suggests that other factors such as the detail of the interventions used and the context of the service in which they were used were also important.

A recently published pilot of a QIC for stroke care in the US showed improvements in care, but this study was limited by problems of data quality and selection of emergency medical services on the basis of use of electronic records and automated quality data [11]. Overall, our findings support the notion of engagement, information, and infrastructure as critically important for success [28].

### Challenges

There were contextual challenges at local and national levels. Local teams sometimes found it difficult to implement ideas because of organizational barriers. Some QI teams felt there was insufficient time to undertake the work. Nationally, there were challenges in sustaining engagement from some participating trusts, partly because of organizational and personnel changes and also due to competing pressures, which led one trust to leave the program.

Staff motivation waxed and waned in response to factors outside the control of the project (*e.g.*, resources, localized restructuring, changes to shift rosters and trust performance). One trust sustained staff engagement by resourcing collaborative members’ work (through time off in lieu), whereas in other trusts, clinicians invested their time on a voluntary basis. Although many trusts achieved significant improvements despite these constraints, services should provide realistic time and resource for improvements to be achieved, rather than relying on goodwill.

### Spread

We adopted a variety of methods to communicate progress of the ACSQI to CEOs and senior managers and to feed back the effects of QI approaches. We fed data back to organizations and shared success stories in a way that was meaningful to frontline staff and managers. Annotated control charts, which showed the outcome of changes implemented, and funnel plots showing how services compared with each other [23] were powerful tools which we would recommend to overcome barriers and obtain wider support for spreading successful interventions [30].

There were differences in the extent, rate and timing of improvement (*i.e.*, early and later adopters) [31], reflecting the scale and challenge of the aim of the QIC. For example, trusts four and eight were later adopters showing later and smaller improvements where these occurred. We found it invaluable to cascade the learning and experience acquired by early adopters through QIF visits and teleconferences and three national meetings. This helped maintain engagement in later adopters or organizations that took longer to show improvements.

We maximized the opportunities for interaction, both formal and informal, between participants. We also used the QI network that we developed to support late adopters, for example, by using staff from early adopter sites to attend and facilitate QI workshops in trusts that were slower to make progress. We would recommend that others wishing to conduct a project on a large scale ensure they develop an effective professional communication and support network [32].

## Conclusions

We found that the specific measures used in this QIC including the use of care bundles as measures, engagement and ownership from front-line staff; application of quality improvement methods (process mapping, plan-do-study-act cycles and annotated control charts), provider prompts and individualized feedback, and opportunities for learning and interaction within and across organizations, helped the collaborative to achieve its aims.

On the basis of this evidence, the care bundles for AMI and stroke have, since this programme, been implemented as part of the national Ambulance Quality Indicator set in England. We are using a multisite comparative case study to explore in greater depth why and how the QIC improved care.

## Competing interests

All authors declare they had no other support from any organization for the submitted work; no financial relationships with any organizations that might have an interest in the submitted work; no other relationships or activities that could appear to have influenced the submitted work.

## Authors’ contributions

ANS had the original idea for the study. ANS, NE and AS were involved in the design and management of the QIC. DS collected the study data. MD undertook statistical analysis and ZD case study data analysis independently of the management of the QIC. All authors were involved in the interpretation and discussion of results. All authors contributed to the writing and review of the various drafts of the report. ANS is guarantor for the study. All authors read and approved the final manuscript.

## Supplementary Material

Additional file 1 Appendices Figure S4–S13. Click here for file

## Data sharing

No additional data available.
