# Supplementary material for: The effect of a national quality improvement collaborative on prehospital care for acute myocardial infarction and stroke in England
Source: Implement Sci. 2014 Jan 23;9:17. doi: 10.1186/1748-5908-9-17 (PMC3904412; doi:10.1186/1748-5908-9-17)
Supplement: Additional file 1 — Appendices Figure S4–S13. [file 1748-5908-9-17-S1.doc]

# Appendix 1 Funnel plots showing care bundle performance across all ambulance trusts for AMI before and at the end of the collaborative (Figures 4-7)

Figure 4 Funnel plot showing care bundle performance for AMI across all ambulance trusts in England before the collaborative began (May 2009)

Figure 5 Funnel plot showing care bundle performance for stroke across all ambulance trusts in England before the collaborative began (July 2009)

Figure 6 Funnel plot showing care bundle performance for AMI across all ambulance trusts in England towards the end of the collaborative (November 2011)

Figure 7 Funnel plot showing care bundle performance for stroke across all ambulance trusts in England towards the end of the collaborative (December 2011)

# Appendix 2 Example annotated control charts for selected trusts (Figures 10-19)

Figure 8 Service 11 Care bundle for AMI

Figure 9 Service 11 Care bundle for stroke

Figure 10 Service 4 Care bundle for AMI

Figure 11 Service 4 Care bundle for stroke

Appendix 3 Control charts for all trusts combined (Figures 20 &21)

Figure 12 All trusts combined care bundle for AMI

Figure 13 All trusts combined care bundle for stroke
